# Oxidative Stress in Neurodegenerative Disorders: A Key Driver in Impairing Skeletal Muscle Health

**DOI:** 10.3390/ijms26125782

**Published:** 2025-06-16

**Authors:** Serena Castelli, Emily Carinci, Sara Baldelli

**Affiliations:** 1 Department for the Promotion of Human Science and Quality of Life, San Raffaele Open University, Via di Val Cannuta, 247, 00166 Rome, Italy; serena.castelli@uniroma5.it (S.C.); emily.carinci@sanraffaele.it (E.C.); 2 IRCCS San Raffaele Roma, 00166 Rome, Italy

**Keywords:** neurodegenerative disease, oxidative stress, skeletal muscle, sarcopenia, atrophy

## Abstract

The fine regulation of antioxidant systems and intracellular production of reactive oxygen species (ROS) is responsible for cellular redox balance. The main organelles responsible for ROS production are mitochondria, and they complete this process through the electron transport chain. These potentially harmful molecules are buffered by enzymatic and non-enzymatic antioxidant systems. Oxidative stress is determined by an imbalance between the production and clearance of ROS in favor of the accumulation of these detrimental species, which generate cellular damage by interacting with macromolecules. In neurodegenerative diseases, oxidative stress has been demonstrated to be a crucial component, both causal and consequential to the disease itself. On the other hand, neurodegeneration disrupts neuromuscular junctions, leading to reduced muscle use and subsequent atrophy. Additionally, systemic inflammation and metabolic dysfunction associated with neurodegenerative diseases exacerbate muscle degeneration. Thus, sarcopenia and atrophy are common consequences of neurodegeneration and play a significant role in these disorders. Regarding this, ROS have been defined as promoting sarcopenia, stimulating the expression of genes typical of this condition. Overall, this review aims to contribute to filling the gap in the literature regarding the consequences at the muscular level of the relationship between oxidative stress and neurodegenerative diseases.

## 1. Introduction

Neurodegenerative diseases are a group of disorders characterized by progressive deterioration and loss of neurons, resulting in both cognitive and motor deficits. Among these conditions, amyotrophic lateral sclerosis (ALS), Alzheimer’s disease (AD) and Parkinson’s disease (PD) stand out. In the first, the degeneration of motor neurons leads to muscle weakness and evolving paralysis; in the second, a constant decline in memory and cognitive functions is observed; finally, the third manifests itself with motor alterations such as tremors and rigidity due to the progressive loss of dopaminergic neurons [1,2,3]. Despite differences in both pathophysiology and symptoms, these diseases share some essential characteristics. One of these is neuronal degeneration, i.e., the gradual decrease in the number of neurons in specific areas of the brain or spinal cord, affecting both the cells responsible for controlling movement—as occurs in ALS and PD—and those involved in cognitive functions, typically compromised in AD [4]. Another common feature is the formation of abnormal protein aggregates, which accumulate inside or near nerve cells. For example, in ALS, deposits of TDP-43 and SOD-1 are observed, while in AD, amyloid-β (Aβ) plaques and tau tangles are present; in PD, instead, the presence of Lewy bodies, made up of α-synuclein (α-syn), is a characteristic feature. These aggregates are able to compromise cellular function and trigger inflammatory processes that further accentuate neuronal degeneration [5,6]. It has been widely demonstrated that chronic oxidative stress is at the basis of the pathogenesis of numerous chronic degenerative diseases, including neurodegenerative ALS, AD and PD [7,8,9].

Redox homeostasis is a fundamental mechanism for cellular life, as it regulates the balance between oxidant species and antioxidant systems. This balance is necessary to ensure the correct execution of numerous biological processes, including energy metabolism, cell signaling, differentiation, immune response and cell survival [10]. Indeed, reactive oxygen species (ROS) and nitrogen species (RNS), mainly produced at the mitochondrial level are also physiologically implicated in signal transduction and cellular homeostasis, on the other hand their excessive accumulation can generate significant molecular damage [11]. Oxidative stress occurs when there is an imbalance in favor of oxidizing agents, which exceeds the capacity of the antioxidant system to neutralize them. This condition involves structural and functional modifications of cellular macromolecules—such as lipid peroxidation, protein carbonylation and DNA mutations—which can compromise cellular integrity and promote pathological processes [12]. It is therefore evident that the fine regulation of redox homeostasis represents a central node for cellular well-being. A deeper understanding of these mechanisms is crucial not only to delineate the molecular pathways involved in cellular physiology but also to identify new therapeutic targets in different pathological conditions where oxidative stress plays a key role.

Indeed, increased oxidative stress represents one of the key pathogenic mechanisms involved in neurodegenerative diseases. These diseases, although presenting distinct clinical and pathophysiological features, share a series of molecular and cellular processes in which the dysfunction of redox homeostasis plays a central role.

Under physiological conditions, the controlled production of ROS and RNS is indispensable for signal transduction, metabolism regulation and immune response. However, in the presence of pathological stimuli or chronic accumulation of stressors, these systems can be overloaded, inducing an imbalance that leads to oxidative damage at the level of lipids, proteins and DNA. Such molecular damage has been implicated in the impairment of mitochondrial function, neuronal dysfunction and synaptic loss, common elements in neurodegenerative diseases [13].

In ALS, for example, evidence suggests that excessive ROS production, combined with impaired antioxidant capacity, contributes to motor neuron degeneration, aggravating cellular damage and accelerating motor decline [14]. Similarly, in AD, oxidative stress promotes abnormal accumulation of β-amyloid protein and the formation of neurofibrillary tangles, exacerbating neuronal damage and synaptic loss [15]. In PD, dopaminergic neuron degeneration is associated with both increased ROS production and reduced antioxidant defenses, phenomena that contribute to the formation of toxic protein aggregates and mitochondrial dysfunction [16].

Skeletal muscle tissue plays a critical role in neurodegenerative diseases, as degeneration of motor neurons and alterations in neural signaling lead to significant loss of muscle function. In ALS, AD and PD, muscles are affected both directly and indirectly, contributing to the debilitating symptoms characteristic of these conditions [17]. In ALS, motor neuron degeneration causes severe muscle atrophy, accompanied by changes in gene expression, protein aggregation, and oxidative stress. These changes further weaken muscle function [18]. In AD, in addition to the known β-amyloid deposits in the brain, accumulations of this protein are also observed in skeletal muscle. These accumulations are associated with mitochondrial dysfunction and reduced muscle oxidative metabolism, contributing to muscle weakness and potentially exacerbating cognitive decline [19,20]. In PD, patients show muscle fiber atrophy, changes in muscle composition, and accumulations of α-synuclein in muscle cells. These changes are related to the typical motor symptoms of the disease and negatively affect the quality of life of patients [21].

This review aims to explore the involvement of muscle tissue in neurodegenerative diseases such as ALS, AD and PD. The main objective is to provide an integrated overview of redox mechanisms and their implications in the processes of muscle atrophy, sarcopenia, mitochondrial dysfunction, protein aggregation and inflammation, examining their involvement in disease progression and symptomatology. Understanding these muscle-related changes will help in the identification of novel drug targets to improve muscle health and patient outcomes.

## 2. Molecular Mechanisms in Redox Homeostasis

### 2.1. Role of Pro-Oxidant Enzymes in Neurodegenerative Disorders

Free radicals are species that possess one or more unpaired electrons and are therefore able to react with other molecules to return to a stable condition. If they react with organic molecules such as proteins, lipids or DNA, then they can cause serious cellular damage. The free radicals of oxygen are superoxide anion radicals (O_2_•^−^) and hydroxyl radicals (•OH), whereas hydrogen peroxide (H_2_O_2_) is a non-radical form of ROS [22]. These species are formed naturally in the cells during the normal cellular pathways, and in specific cells like macrophages, they can be exploited to fight infections. These species can also be formed following exposure to exogenous factors such as cigarette smoke or UV radiation, among others. Specialized antioxidant enzymatic systems are responsible for neutralizing excess ROS and maintaining cellular redox balance, preventing oxidative damage to cells and tissues.

One of the enzymes capable of producing reactive oxygen species, and specifically O_2_•^−^, is the family of nicotinamide adenine dinucleotide phosphate oxidase or NADPH oxidase (NOX). This enzyme is present in cell membranes and in phagosomes. This family of proteins is essential for host defense, signaling, and cell differentiation [23].

The NOX protein family consists of 7 members: NOX1, NOX2, NOX3, NOX4, NOX5, and dual oxidases DUOX1 and DUOX2. This family belongs to the ferric reductase superfamily and all enzymes that are part of it have a rather conserved structure. The catalytic core is composed of two domains, the dehydrogenase (DH) domain and the transmembrane (TM) domain, consisting of six transmembrane α-helices containing two binding sites for heme groups. The DH domain is cytosolic and hosts the binding sites for the coenzymes FAD at the N-terminal and NADPH at the C-terminal. Both heme groups and coenzymes bind to the protein non-covalently [24,25]. The various NOX isoforms, except NOX4, are not active in monomeric form, but become active when they bind to other proteins or factors. NOX5, DUOX1 and DUOX2 are activated by intracellular calcium (Ca^2+^) signaling. NOX5 directly binds Ca^2+^ through its EF-hand domains. DUOX1 and DUOX2 require calcium and the DUOA proteins for activation. To be activated, NOX2 requires the integral membrane proteins gp91^phox^ and pp22^phox^. The activation of NOX2 requires that the regulatory subunit p47^phox^ be phosphorylated so that it can interact with the p67^phox^ subunit. This causes the translocation of the cytosolic subunits to the membrane and allows the activation of NOX2 thanks to the interaction of these subunits with cytochrome b558 (gp91^phox^ and p22^phox^). In addition to the catalytic core, NOX2 also contains the transmembrane flavocytochrome b588, the cytosolic subunits and the G-proteins Rac1 and Rac2 in monocytes and neutrophils, respectively. The assembly of the active enzyme occurs only in the presence of microbial infections, and this ensures the production of the O_2_•^−^ [26]. The formation of the O_2_•^−^ mediated by NOX involves a passage of electrons from NADPH to FAD, which is reduced to FADH2. FADH2 is able to give one electron at a time to the heme groups, forming two superoxide anions. NOX proteins are essential for defending the body against infections, being capable of producing ROS. However, their malfunction is often associated with various types of chronic diseases, including cardiovascular diseases, neurodegenerative diseases and cancer [27]. The role that NOX has in neurodegenerative diseases is crucial, as our brain consumes about 20% of the O_2_ available in the body, and this high presence of O_2_ in the brain causes a high production of ROS. The most present isoform in the brain is NOX2 followed by NOX1 and NOX4 [24].

The role that ROS and specifically O2•^−^ have in the progression of neurodegenerative diseases is related to their ability to oxidize proteins and lipids and cause DNA. In AD, it has been seen that the accumulation of ROS can alter vascular regulation, compromising cerebral perfusion, thus preventing flow towards regions of the brain that undergo neuronal death [28].

In addition to NOX, another key enzyme in ROS production is myeloperoxidase (MPO). MPO is an enzyme belonging to the heme peroxidase superfamily present in azurophil granules of neutrophils and monocytes. MPO is important for antimicrobial activity but a high MPO’s activity is related to oxidative stress [29]. MPO is a homodimer in which the two monomers are both catalytic and are joined by a disulfide bridge. The interface between the two monomers presents glycosylations as post-translational modifications of the amino acids, to ensure the stability of the protein even in the presence of proteases naturally present in lysosomes. A Ca^2+^ ion is also present which stabilizes the protein structure but has no catalytic function. The core is formed by a heme group with Fe^3+^ in its basal state. The catalytic cycle of MPO involves the binding of the oxygen of H_2_O_2_ (derived from the dismutation of O_2_•^−^–by SOD-which in turn is produced by NOX) to the Fe^3+^ which will be further oxidized to Fe^4+^. At the same time, H_2_O is released and the oxygen bound to the iron of the heme will form a radical on the porphyrin ring giving the highly reactive compound **1**. From compound **1**, two cycles can be undertaken to ensure the return of the enzyme to its basal state. The halogenation cycle in which compound **1** reacts with halogen ions such as Cl^−^ or Br^−^ to produce hypochlorous acid (HOCl) or hypobromous acid (HOBr) with antimicrobial action. The peroxidase cycle is instead undertaken when the levels of H_2_O_2_ are high, and the quantity of halogens present is insufficient to complete the halogenation cycle. In this case, to reconvert the enzyme to its basal state, the radical on the porphyrin ring is neutralized by other molecules generically indicated with RH_2_, of both organic and inorganic nature (Tyr, Trp, Thiols, Ascorbate, O_2_•^−^, NO), capable of oxidizing and assuming the radical form. This form is called compound **2**, with the iron still present as Fe^4+^, which must react with another RH_2_ molecule to return to its basal state [30].

MPO is implicated in neurodegenerative disease and sarcopenia. In neurodegenerative disease the role of MPO is crucial because it produces HOCl and HOCl and several HOCl-generated markers such as 3-chlorotyrosine have been identified in AD patients [31].

In addition to these enzymes, other enzyme belonging to the pro-oxidant systems is Xanthine oxidase (XOR). This enzyme is essential for the catabolism of purine nucleotides, in fact, it catalyzes the oxidation of hypoxanthine to xanthine, and of xanthine to uric acid. Its mechanism of action, however, involves the reduction of O_2_ to O_2_•^−^ which will then be converted to H_2_O_2_. The accumulation of H_2_O_2_ is related to ND and AD pathogenesis [32]. XOR is an oxidoreductase belonging to the molybdoflavoenzyme family. It is a 300 kDa homodimer, and each subunit has 3 main domains. The N-terminal domain contains two non-identical iron–sulfur centers (2Fe/S), the intermediate domain has a FAD+ that acts as a cofactor, while the C-terminal domain contains a molybdenum (Mo) that acts as a cofactor. The catalytic domain is the one that has the Mo. When the substrate (xanthine or hypoxanthine) binds to the molybdenum center, the Mo^6+^ is reduced to Mo^4+^, oxidizing the substrate, and the electrons pass from the Mo to the Fe/S centers up to the FAD. From FADH+H+ the electrons pass to O_2_ which is converted to O_2_•^−^ and H_2_O_2_ [33].

### 2.2. Role of Anti-Oxidant Enzymes in Neurodegenerative Disorders

While enzymes such as NADPH oxidase, MPO and xanthine oxidase play a key role in the production of ROS under physiological and pathological conditions, excessive and uncontrolled ROS generation can lead to oxidative damage and cellular dysfunction. To counterbalance this potentially harmful oxidative burden, the organism relies on a tightly regulated network of antioxidant defense mechanisms aimed at preserving redox homeostasis [34].

Superoxide dismutases (SODs) are oxidoreductases using various metals as cofactors in the active site. Their function is to convert O_2_•^−^ into H_2_O_2_ and O_2_. SODs are classified based on the metal present in their active site into three main isoforms in the human body: SOD1, containing copper and zinc (Cu/Zn); SOD2, containing manganese (Mn); and SOD3, which also contains copper and zinc. SOD1 is primarily localized in the cytosol, SOD2 is mainly found in the mitochondria, and SOD3 is localized in the extracellular fluids [35]. Their catalytic core is highly conserved, although the various human SODs are encoded by different genes. SOD1 is a homodimer, while SOD2 and SOD3 function as tetramers. SOD2 has homology to Fe-SOD found in prokaryotes and is altered under conditions of neuroinflammation in diseases such as AD and PD [36]. Among the SODs, SOD1 is the most extensively studied isoform and is particularly associated with ALS. Indeed, a largely used mouse model in ALS is that mutated on SOD1-G93A, which is characterized by the canonical neurodegenerative symptoms of ALS [37].

SOD1 exhibits a highly conserved amino acid sequence. This remarkable conservation is also reflected in the enzyme’s three-dimensional structure, which remains virtually unchanged across different species. Such preservation highlights the crucial evolutionary importance of SOD1: Its catalytic efficiency is so optimized that significant structural modifications have not been required during the evolutionary adaptation of organisms. In addition to the primary sequence, the secondary, tertiary and quaternary structures of SOD1 are also highly conserved. The enzyme consists of two dimers that associate to form the functional unit; each dimer is mainly composed of secondary structures arranged in a β-barrel fold (i.e., β-sheets organized into a barrel-like structure), accompanied by α-helical elements. A binding region stabilized by a disulfide bond is present between the two subunits forming the dimer. Moreover, the metal cofactors Cu and Zn are coordinated by the enzyme and are located externally relative to the β-barrel.

Superoxide dismutation by SOD1 occurs in two redox steps involving copper in the active site and involves the sequential binding of two superoxide molecules with the final release of H_2_O_2_ and O_2_. In the basal state, copper is in the oxidized form Cu^2+^ and coordinates to three histidine (His) residues, a bridge His that also binds zinc, and an H_2_O. When copper is in this configuration, an O_2_•^−^ enters the active site, replacing the water molecule that coordinated copper. Superoxide binds directly to copper, which is then reduced from Cu^2+^ to Cu^+^, while O_2_•^−^ is oxidized to O_2_ which is released. During this process, protonation of the bridge histidine causes the bond between this histidine and the reduced copper to be lost. Subsequently, the second superoxide molecule enters the catalytic site, which initially does not bind directly to copper but is coordinated by adjacent residues, the histidine bridge and a water molecule. This O_2_•^−^ withdraws a proton from the histidine bridge and two electrons from the copper Cu^+^, which is oxidized again to Cu^2+^. Consequently, the superoxide is reduced to H_2_O_2_, the histidine bridge loses the proton, and the copper returns to the basal oxidized state Cu^2+^. Finally, the hydrogen peroxide exits the active site, aided by the water molecule that returns to its initial position, which is essential for the coordination of copper [38].

Catalase (CAT) is a heme-containing peroxidase predominantly localized in the peroxisomes of eukaryotic cells and in certain prokaryotic species. It catalyzes the dismutation of H_2_O_2_ generated by cellular metabolic processes and as a byproduct of SOD activity into H_2_O and O_2_. By mitigating H_2_O_2_-induced oxidative stress, catalase plays a critical role in maintaining redox homeostasis, contributing to neuroprotection and the modulation of aging-related processes [39]. There are three isoforms of catalase, two containing heme and one containing Mn. In humans, the heme-containing catalase is present, and is active in the tetrameric form [40]. The mechanism by which CAT decomposes H_2_O_2_ into H_2_O and O_2_ involves several steps. H_2_O_2_ binds to Fe^3+^ in the active site of the enzyme, after which the enzyme is oxidized and H_2_O_2_ is reduced to H_2_O. This reaction transforms ferric iron into an intermediate with an iron-oxo species (Fe=O) and a cation on the porphyrin radical; this form is known as Compound **1**. The highly reactive Compound **1** reacts with another molecule of H_2_O_2_, which cleaves the bond between the two oxygens of the molecule, releasing O_2_ and H_2_O. The reduction of Compound **1**, which is essential for restoring the catalytic site to its initial state, occurs thanks to a distal histidine (His=) that acts as an acid–base catalyst and allows the transfer of hydrogen [41] (Table 1).

This table provides an overview of the main enzymes discussed, categorized by their roles in ROS production and anti-oxidant defenses. Pro-oxidant enzymes such as NADPH oxidase, myeloperoxidase, and xanthine oxidase generate reactive species that contribute to microbial killing, inflammatory responses and cell differentiation but can also cause cellular damage. Conversely, anti-oxidant enzymes including SODs and catalase catalyze the detoxification of ROS, maintaining cellular redox balance and protecting cells from oxidative stress.

## 3. Metabolic Dysfunctions of Skeletal Muscle in Neurodegeneration

### 3.1. Role of Mitochondria in Skeletal Muscle Dysfunction Typical of Neurodegeneration

Skeletal muscle metabolic dysfunction has increasingly been recognized as a contributing factor in the pathogenesis and progression of neurodegenerative diseases. Rather than being a passive target of neural decline, skeletal muscle actively influences neurodegenerative processes through metabolic and endocrine pathways [18,42]. Sarcopenia, the leading cause of loss of independence and reduced quality of life in the elderly, is frequently observed in patients with neurodegenerative disorders such as AD, PD, and ALS [43,44]. Although sarcopenia has traditionally been considered a consequence of neurodegeneration, primarily due to impaired mobility, emerging literature suggests that sarcopenia may also act as a contributing factor in the onset of neurodegeneration. Clinically, sarcopenia has been detected in terms of leg lean mass and hand grip strength prior to the onset of neurodegeneration, demonstrating potential as a predictive marker for the neurodegenerative disorder itself. This aspect is particularly important both for enabling the earliest possible intervention in neurodegeneration and because the diagnosis of sarcopenia is relatively quick and straightforward to perform. Hand grip strength is widely used for this purpose and is measured using a hand dynamometer. Muscle mass assessment can be carried out using whole-body dual-energy X-ray absorptiometry (DXA). The appendicular skeletal muscle mass index (ASMI) is generally calculated by dividing the sum of lean mass in both upper and lower limbs by the square of the height (lean mass of bilateral upper and lower extremities/[height]^2^). Reduced muscle mass was defined as ASMI < 7.0 kg/m^2^ in males and <5.4 kg/m^2^ in females. Although reference values differ between males and females, no association has been found between sex and the development of sarcopenia. Instead, sarcopenia is primarily associated with aging, as muscle loss occurs physiologically with increasing age [44].

Moreover, the progressive decline in muscle mass leads to a reduced secretion of myokines by skeletal muscle, potentially disrupting neuroprotective signaling pathways [45]. Among the principal myokines produced by skeletal muscle are IL-6, IL-7, IL-8, irisin, myostatin and various chemokines [46]. These molecules play a key role in sustaining the muscle–brain axis, and dysregulation of their production can lead to cerebral dysfunction through both paracrine and autocrine mechanisms. In this context, it has been observed that reduced myokine secretion by skeletal muscle results in increased production of pro-inflammatory cytokines by astrocytes and microglia, contributing to memory impairments. Conversely, oxidative stress promotes the production of pro-inflammatory cytokines within muscle tissue, contributing to muscle loss and the development of sarcopenia. These cytokines are also capable of crossing the blood–brain barrier, thereby influencing muscle–brain communication [47].

In addition to being a significant prognostic factor in neurodegenerative diseases, sarcopenia also constitutes a risk factor, as the decline in muscle mass has been shown to influence susceptibility to AD and other forms of dementia [44].

The loss of muscle mass that characterizes sarcopenia is also associated with a rearrangement in muscle quality, leading to reduced contractile strength. This decline in muscle function results in decreased mobility in affected individuals and may precede the clinical onset of neurodegenerative pathology [48]. It has indeed been observed that skeletal muscle undergoes metabolic rewiring before the onset of neurodegenerative symptoms and sarcopenia, leading to increased energy consumption regardless of physical activity. To meet this elevated energy demand, muscle tissue mobilizes lipid reserves as a primary energy source [42,49]. Since fatty acids are metabolized in the mitochondria and these organelles are the cell’s powerhouse, mitochondrial metabolism plays a crucial role in the development of sarcopenia and the muscle rearrangement observed in neurodegenerative diseases.

Skeletal muscle is composed of a mixed type of fiber. Muscle fiber contains repeating units of actin and myosin filaments forming sarcomeres, responsible for muscle movement. The contractile capacity of a muscle fiber relies on the intrinsic ATP hydrolysis activity of the myosin heavy chain (MyHC). Based on the expression of sarcomeric MyHC isoforms, skeletal muscle can be classified into type I (or slow-twitch) fibers, expressing MyHC-I, and type II (or fast-twitch) fibers, expressing MyHC-IIa, MyHC-IIb and MyHC-IIx [10]. Beyond MyHC expression, fibers are also different for metabolic features directly associated with the type of contraction performed, namely, a slow-speed contraction for slow-twitch type I myofibers and a rapid contraction for the fast-twitch type IIB/X fibers. Type I fibers are the most fatigue resistant, highly vascularized and show high levels of mitochondria and myoglobin. They mainly rely on lipid catabolism and oxidative phosphorylation for energy production. Type II fibers are easily fatigued and mainly depend on glycolysis for energy supply. Type IIA fibers show intermediate features [49,50]. The metabolism and the type of contraction that the fiber can perform are strictly correlated, and even the sole increase in mitochondrial metabolism determines a variation in the type of fiber, with the induction of a type I phenotype [51]. It has been observed that during neurodegenerative diseases muscle fibers tend to be more oxidative, therefore more similar to type I. In this regard, it has been demonstrated that N-acetylaspartate (NAA) released following neuronal rupture typical of neurodegenerative diseases, beyond having an action at the central level, reducing the pro-inflammatory microglial response [52], at the peripheral level induces a switch from type II fibers to type I fibers of muscle [51].

The debate is still open on the definition of this effect as preceding or consequent to neurodegeneration [42,53].

This transition from fast to slow fibers also characterizes sarcopenia and it is related to a metabolic rewiring [54]. A proteomic analysis conducted on age-related sarcopenic muscles revealed a shift in the expression of genes associated with the contractile apparatus toward those characteristics of slow-twitch fibers [55]. Furthermore, senescent muscles exhibit a predominantly oxidative metabolism, accompanied by a marked downregulation of glycolytic gene expression. This metabolic shift is also associated with the higher susceptibility of glycolytic fibers to muscle atrophy, and thus, it became a protective mechanism [54]. The enhancement of energy metabolism in skeletal myocytes has been demonstrated to be protective against sarcopenia and avoid reduction in myotubes’ diameter [56]. Indeed, fast/glycolytic fibers tend to express higher levels of pro-atrophic genes compared to slow/oxidative fibers, also promoting the expression of pro-inflammatory genes such as NF-κB [57]. An additional advantage associated with the fiber type transition lies in the greater abundance of satellite cells in slow-twitch fibers, which enhances their capacity to respond to external stimuli. Several signaling pathways mediate the fast-to-slow fiber type shift, including PGC-1α signaling, calcium–NFAT/MEF2 and calcium–CaMK/MEF2 signaling, MAPK signaling, WNT signaling, as well as regulatory elements such as microRNAs, long non-coding RNAs (lncRNAs), and other transcription factors. Among these, PGC-1α exerts a protective role against atrophic stimuli and acts synergistically with calcineurin/nuclear factor of activated T cells (NFAT) signaling to maintain the oxidative phenotype of myofibers [57]. In this context, PGC1α promotes mitochondrial biogenesis and facilitates the shift toward oxidative metabolism. Mitochondrial mass loss is, in fact, a hallmark of aging and sarcopenia. Moreover, PGC1α plays a crucial role in maintaining the neuromuscular junction and the innervation of myofibers, both of which are progressively lost during muscle aging and in neurodegenerative conditions [58]. Although oxidative metabolism is beneficial for muscle fiber fitness and exerts protective effects against sarcopenia and neurodegenerative diseases, the muscle mass loss characteristic of these conditions is associated with increased proteolysis and an imbalance in protein turnover, driven by elevated intracellular ROS levels. Mitochondria are the primary source of ROS in the cell, and an enhanced rate of mitochondrial metabolism—if not adequately counteracted by antioxidant systems—can lead to oxidative stress. Skeletal muscle is inherently more prone to oxidative stress compared to other tissues, both because it is a post-mitotic tissue and generally consumes a high amount of oxygen. Oxidative stress, together with glucocorticoids and pro-inflammatory cytokines, constitutes a major stimulus for protein degradation [59]. For this reason, several ROS-responsive factors are involved in the promotion of sarcopenia and muscle atrophy. Among these are MAPKs such as p38 and ERK, which, upon activation by increased intracellular ROS levels, regulate the proteolytic response by promoting protein catabolism, thereby contributing to sarcopenia and atrophy. Specifically, p38 has been associated with the upregulation of Atrogin-1, a major muscle-specific E3 ubiquitin ligase involved in protein degradation during muscle atrophy [60]. ERK has been identified as a key mediator of the atrophic response to indoxyl sulfate in C2C12 myotubes, promoting the upregulation of Atrogin-1 [61].

**Figure 1:**
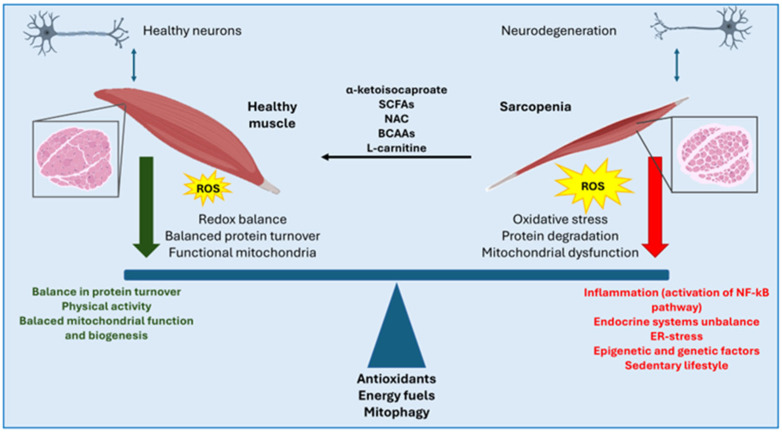
The balance between a healthy muscle and a sarcopenic one is associated with antioxidant capacities, the different types of energy fuels used and the mitochondrial homeostasis. Moreover, additional pathways may be involved in the pathogenesis and progression of sarcopenia associated with neurodegeneration, including inflammatory pathways (e.g., NF-κB), hormonal alterations, dysregulation of mitochondrial function and biogenesis, endoplasmic reticulum stress (ER-stress), a sedentary lifestyle, as well as genetic and epigenetic factors. Among the therapeutic approaches that have shown potential in reversing and preventing sarcopenia are the use of α-ketoisocaproate, and more broadly, short-chain fatty acids (SCFAs), branched-chain amino acids (BCAAs) and L-carnitine.

Atrophy is a condition observed in patients with neurodegenerative diseases, characterized by muscle mass loss and reduced contractile responsiveness. At the molecular level, this response in muscle fibers is driven by a massive activation of protein degradation pathways, resulting in a net reduction of cellular protein content. Similar to sarcopenia, atrophy is also associated with a metabolic shift toward oxidative metabolism and a decrease in myotube diameter [62]. In both sarcopenia and atrophy, which are phenotypically very similar, oxidative stress impairs myotube fitness by reducing the differentiation capacity of satellite cells and, more broadly, diminishing the muscle’s ability to respond to external insults [63]. Redox homeostasis, therefore, plays a key role in reducing myotube damages in neurodegenerative diseases. In this context, the muscle cell’s antioxidant defenses are crucial in regulating the proteolytic response by modulating intracellular ROS levels (Figure 1).

**Figure 2:**
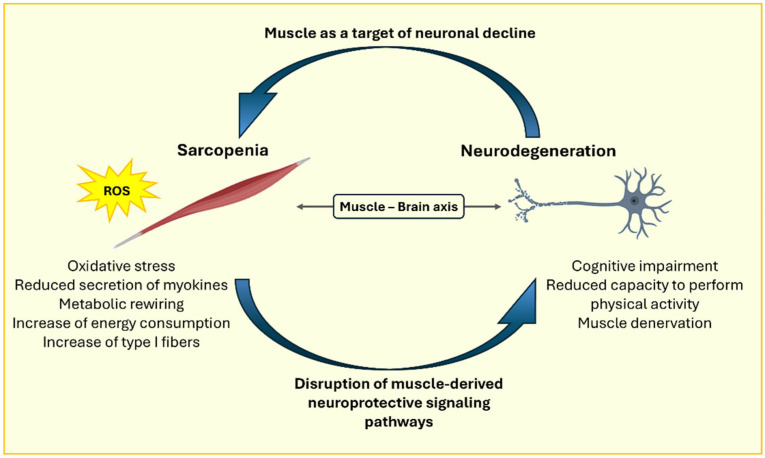
Representative image of the bidirectional relationship between sarcopenia and neurodegeneration.

Mitochondrial dysfunction is generally associated with the worsening of symptoms in several neurodegenerative disorders, including AD, where enhanced mitochondrial function in vivo has been linked to reduced amyloid levels. Epidemiologically, reduced mitochondrial functionality of skeletal muscle has been associated with cognitive impairment and has been proposed as a predictive risk factor for dementia. Based on this evidence, skeletal muscle mitochondrial health has been proposed as a proxy measure for mitochondrial health in other tissues, including the brain. Using 31P magnetic resonance (MR) spectroscopy, the maximal oxidative capacity of the quadriceps muscle was assessed in 649 participants in a longitudinal study, revealing a direct positive association between muscle mitochondrial function and brain health. Indeed, higher skeletal muscle mitochondrial oxidative capacity is associated with preserved brain structure [64]. These findings support the notion that muscle alterations, including oxidative stress and sarcopenia, are not merely consequences of neurodegeneration but may also play an active role in the pathogenesis of neurodegenerative disorders themselves, establishing a bidirectional relationship between the two conditions (Figure 2).

### 3.2. Role of Mitochondria Dynamics in Sarcopenia and Atrophy

As a consequence of the mitochondrial functional alterations, mitochondrial dynamics are modulated during neurodegeneration, leading to a shift in the balance between fusion and fission processes. Mitochondrial fusion and fission mechanisms contribute to mitochondrial homeostasis. Fusion rearranges internal macromolecules within the mitochondrion, promoting the formation of an oxidatively active network. In contrast, fission serves to isolate dysfunctional components, which are subsequently removed via the autophagic pathway of mitophagy [65]. Mitochondrial fusion is regulated by mitofusin 1 and 2 (MFN1 and MFN2). In the context of sarcopenia, MFN2 has been shown to play a key role in mitigating muscle loss. Indeed, MFN2-deficient mice exhibit mitochondrial dysfunction and reduced muscle mass. This regulation is mediated by an increase in ROS following MFN2 downregulation, leading to activation of the HIF1α-BNIP3 pathway, which promotes mitophagy. Moreover, during aging—which is generally characterized by sarcopenia—MFN2 levels are typically reduced [66]. In parallel, Drp1, a protein involved in mitochondrial fission, is more strongly associated with reduced muscle health and impaired mitochondrial function. Overexpression of Drp1 in mice has been shown to negatively affect both mitochondrial and muscle function [67].

Mitophagy is a degradation pathway targeting dysfunctional or aged mitochondria through the autophagy–lysosome machinery. It generally follows mitochondrial fission, which serves to isolate the damaged portion of the network for removal. Mitophagy has been shown to be critical for maintaining skeletal muscle health, as defects in this pathway are associated with increased expression of atrophic markers [68]. In this context, enhancing mitophagy has been explored as a therapeutic strategy to support muscle function and prevent sarcopenia [69].

### 3.3. Therapeutical Approaches to Sarcopenia/Atrophy

Several therapeutic approaches for sarcopenia leverage the ability of exogenous antioxidants to attenuate the atrophic response either by reducing oxidative stress or by inhibiting ROS-responsive signaling pathways (Figure 1). For instance, α-ketoisocaproate has been shown to reduce the atrophic response in vitro in C2C12 myotubes by inhibiting the MAPK signaling pathway activated in response to elevated ROS levels [70]. Another example is the therapeutic approach involving carnitine supplementation, which, in addition to exerting anti-catabolic effects, also plays an antioxidant role in skeletal muscle [62,71]. N-acetylcysteine (NAC), a potent antioxidant, has been reported to reverse exogenously induced atrophy by downregulating the expression of proteolytic genes [61]. This dual nature confers on mitochondria an ambivalent role. On one hand, they support muscle fiber resistance and endurance; on the other, they contribute to increased intracellular ROS levels and promote oxidative stress. Another example of treatment is that involving short-chain fatty acids supplementation, such as butyrate, which has been shown to reduce oxidative stress and improve muscle atrophy [72]. The primary source of short-chain fatty acids (SCFAs) is the gut microbiota, which is often dysregulated in neurodegenerative diseases. The dysbiosis observed in these conditions is responsible for intestinal inflammation mediated by lipopolysaccharides (LPS), which activate the NF-κB signaling pathway. At the muscular level, LPS can bind to Toll-like receptor 4 (TLR4), triggering the activation of the ubiquitin–proteasome system, thereby promoting sarcopenia. Thus, the gut microbiome also plays a key role in the systemic relationship between muscle and brain. Several studies have highlighted alterations in microbiome-derived metabolites in cases of sarcopenia. A substantial depletion of the gut microbiota through antibiotic treatment has been shown to reduce muscle strength in mice, as well as the extensor digitorum longus muscle fatigue index in an ex vivo contractile test.

Although the mechanisms underlying this gut–muscle cross-talk are not yet fully understood, it appears to affect glycogen availability within the muscle, a critical energy reserve particularly important during endurance-demanding activities. Moreover, alterations in the gut microbiome are also associated with increased inflammation, particularly at the intestinal level, which is reflected in elevated inflammatory markers within skeletal muscle, as well as markers related to protein turnover and mitochondrial metabolism [73].

Given the symptomatic and molecular complexity of sarcopenia, the therapeutic approach is generally multifaceted, involving both lifestyle interventions and pharmacological agents. Among the latter, many compounds used in treatment exhibit antioxidant properties—such as coenzyme Q10 and NAC—or support muscle mass and reduce fatigue, including L-carnitine and branched-chain amino acids (BCAAs) [62,73]. The main limitation of the clinical use of antioxidants lies in their reduced ability to reach effective concentrations in vivo. This has also driven the development of parallel strategies aimed at enhancing endogenous antioxidant activity. Another important aspect to consider is the inter-individual variability, among which age stands out as a key factor, given that endogenous antioxidant defenses decline with aging. Regarding the therapeutic approach related to lifestyle, physical exercise has demonstrated efficacy both in reducing oxidative stress and in remodeling muscle fiber metabolism, promoting increases in muscle mass and endurance.

## 4. Oxidative Stress in Skeletal Muscle Dysfunction Associated with Neurodegenerative Diseases

In the context of neurodegenerative diseases, the role of muscle tissue emerges as a key element not only in clinical manifestations but also in therapeutic strategies. The impairment of the central and peripheral nervous system significantly alters neuromuscular transmission, leading to a progressive deterioration of muscle function. In conditions such as ALS, AD and PD, skeletal muscle is directly or indirectly involved, contributing significantly to the associated disability [74]. In the case of ALS, the degeneration of motor neurons originating at the cerebral and spinal level determines a marked muscle atrophy, accompanied by increasing weakness, which compromises the patient’s autonomy and limits the execution of daily activities [75,76]. Although AD is mainly identified for cognitive decline, several studies report a correlation with age-related sarcopenia, a condition that reduces muscle mass and strength, increasing the risk of falls and promoting loss of mobility [76]. Similarly, in PD, the decline in dopamine levels affects motor function, causing stiffness, tremors and slowness of movement (bradykinesia), all factors that hinder balance and walking [77].

### 4.1. Role of Oxidative Stress in ALS Skeletal Muscle

In ALS, progressive muscle atrophy is a direct consequence of impaired nerve transmission due to the degeneration of motor neurons. The pathological process originates at the level of lower motor neurons, which, when they fail, interrupt the normal functioning of neuromuscular junctions (NMJs)—highly specialized synaptic structures that mediate communication between motor neurons and muscle cells. The loss of neuronal input prevents muscles from contracting adequately, resulting in a progressive reduction in both strength and muscle mass. This deterioration initially manifests itself with an evident narrowing of muscle fibers (atrophy), which in a more advanced stage may culminate in their complete disappearance [78]. In ALS, muscle atrophy results from a combination of disrupted cellular processes that regulate protein turnover and cell survival. A central component in this degradation machinery is the ubiquitin–proteasome system (UPS), which ordinarily facilitates the clearance of misfolded or damaged proteins [79]. In ALS, there is a notable upregulation of muscle-specific E3 ubiquitin ligases, particularly MuRF1 and atrogin-1/MAFbx, which tag structural muscle proteins for breakdown [80]. This heightened activity of the UPS contributes to accelerated degradation of muscle tissue and exacerbates wasting [81]. Parallel to this, the autophagy–lysosome pathway (ALP)—another key proteolytic system—exhibits abnormal activation in ALS. Autophagy, a catabolic process involving the lysosomal recycling of cellular components, becomes dysregulated in the affected musculature. Instead of maintaining cellular homeostasis, overactive autophagy leads to excessive degradation of essential proteins and organelles, thus amplifying muscle degeneration [82,83]. Moreover, apoptotic signaling plays a substantial role in the loss of muscle mass in ALS. Elevated expression of pro-apoptotic mediators such as Bax, alongside reduced levels of protective proteins like Bcl-2, contributes to the execution of apoptosis within muscle tissue. These apoptotic mechanisms are frequently triggered by cellular stressors, including oxidative damage and mitochondrial dysfunction, ultimately leading to the systematic disassembly and clearance of muscle cells [84].

In ALS, muscles undergo cycles of denervation and partial reinnervation, with early compensatory repair failing over time due to ongoing motor neuron loss. The disease preferentially affects fast-twitch muscle fibers, altering muscle function and contributing to weakness and fatigue. In the context of ALS, numerous studies have demonstrated that oxidative damage disproportionately affects fast-twitch fibers, contributing to their early and pronounced degeneration [85,86]. The heightened sensitivity of these fibers to redox imbalance underscores the pivotal role of oxidative stress in the selective vulnerability observed in ALS and other neuromuscular disorders.

In ALS, impaired mitochondrial function is a key contributor to muscle degeneration. In affected muscle fibers, mitochondria often display significant structural distortions—such as swollen, fragmented inner membranes—that drastically reduce their capacity to generate ATP [87]. This failure in energy production is accompanied by an overproduction of ROS, which intensifies cellular oxidative stress [88]. The oxidative imbalance in ALS muscle is evident from elevated levels of markers like lipid peroxides and oxidized proteins, signifying extensive oxidative damage [89]. This hostile environment not only disrupts energy metabolism but also activates catabolic pathways that break down muscle proteins, while simultaneously hindering the muscle’s natural repair processes. Over time, these effects contribute significantly to the progression of muscle atrophy in ALS [90]. Moreover, the oxidative stress in muscle tissue interferes with redox-sensitive signaling pathways, further compounding the detrimental effects on muscle cell function. This intricate interplay between mitochondrial damage, bioenergetic failure, and oxidative stress creates a vicious cycle that accelerates the deterioration of muscle tissue in ALS.

### 4.2. Role of Oxidative Stress in AD Skeletal Muscle

AD is a complex neurodegenerative disease characterized by several interconnected pathogenic mechanisms. The main ones include the extracellular accumulation of amyloid-beta (Aβ) plaques, the intracellular formation of neurofibrillary tangles composed of hyperphosphorylated tau protein, chronic inflammation mediated by microglia and astrocytes, oxidative stress, and mitochondrial dysfunction [91]. These processes collectively contribute to the neuronal degeneration and cognitive decline observed in AD patients. The abnormal production and accumulation of Aβ results from the improper cleavage of amyloid precursor protein (APP) by β- and γ-secretases. This process generates Aβ monomers that tend to aggregate to form toxic oligomers and, subsequently, extracellular senile plaques. These plaques are associated with synaptic dysfunction and neuronal death [92]. Furthermore, tau protein, normally involved in stabilizing neuronal microtubules, undergoes hyperphosphorylation in AD, losing its function and aggregating into intracellular neurofibrillary tangles. These tangles disrupt axonal transport and contribute to neuronal degeneration [93].

In addition, mitochondrial dysfunction and imbalance between ROS and antioxidant defenses have been frequently observed in AD patients, which further contribute to the development of oxidative stress and neuronal energy impairment [93]. All these pathogenic processes are interconnected: Aβ accumulation can induce abnormal tau phosphorylation; neuroinflammation can increase Aβ production and tau phosphorylation; oxidative stress can exacerbate both pathologies. This complex interaction contributes to the progression of AD.

The role of muscle tissue in AD is a developing area of research that could provide new insights into the disease mechanisms and potential therapeutic interventions. Alterations in muscle tissue in patients with AD are multifactorial and can be attributed to various pathophysiological mechanisms. One of the key mechanisms is mitochondrial dysfunction, which causes a reduction ATP production and increased oxidative stress. Muscle cells with malfunctioning mitochondria are unable to maintain efficient energy metabolism, resulting in muscle weakness and fatigue. The inability of mitochondria to generate appropriate energy disrupts normal muscle function and contributes to the overall decline in physical capabilities observed in AD patients [94]. Additionally, the accumulation of Aβ has also been observed in skeletal muscles. This toxic peptide can interfere with muscle function through various mechanisms, including the induction of oxidative stress, ER dysfunction, and the activation of pro-apoptotic pathways. The accumulation of Aβ in muscles may directly contribute to the muscle mass loss and muscle dysfunction observed in AD patients. Therapies targeting Aβ may benefit both neurological and muscle systems due to their pathological overlap [95].

### 4.3. Role of Oxidative Stress in PD Skeletal Muscle

PD is a progressive neurodegenerative disorder characterized by the loss of dopamine-producing neurons in the pars compacta of the substantia nigra of the midbrain [96]. Neuronal decline lowers dopamine levels in the striatum, disrupting motor control and causing symptoms such as resting tremors, muscle rigidity, and slowed movements [97,98]. The development of PD is complex and involves both genetic predispositions and environmental influences. Approximately 10–15% of cases are familial, highlighting a strong genetic link. Mutations in several genes, including SNCA (encoding α-synuclein), LRRK2, PARK2, PARK7, and PINK1, can interfere with normal cellular operations and drive neurodegeneration [99,100,101,102]. A hallmark of the disease is the formation of Lewy bodies, abnormal cytoplasmic inclusions consisting primarily of aggregated α-synuclein. Normally, α-synuclein helps regulate synaptic function and neuronal adaptability; however, under pathological conditions, it folds abnormally and aggregates into toxic forms that disrupt synaptic communication and contribute to neuronal death.

Mitochondrial dysfunction and oxidative stress are key factors in the development of PD. When mitochondria are impaired, they generate excessive ROS, triggering oxidative stress that damages cellular macromolecules and leads to neuronal death [103,104]. Additionally, disruptions in autophagy—a critical process for recycling defective proteins and organelles—result in the accumulation of misfolded proteins and cellular debris [105]. Similarly, malfunction of the ubiquitin–proteasome system, another essential protein degradation pathway, contributes to the buildup of toxic proteins like α-synuclein [106].

**Figure 3:**
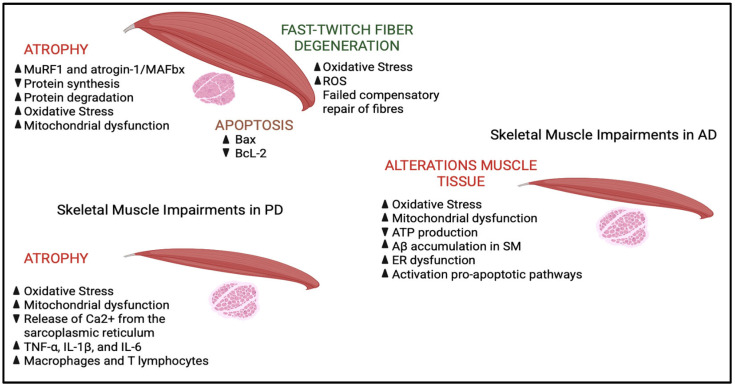
Schematic view of skeletal muscle impairments in ALS, AD and PD. In ALS, muscle atrophy is caused by the degeneration of motor neurons, which impairs neuromuscular transmission and reduces muscle spreading. At the molecular level, there is a marked activation of the ubiquitin–proteasome system, with an increase in muscle-specific E3 ligases, such as MuRF1, which promote the degradation of structural proteins. In parallel, the autophagy–lysosome system becomes overactive, further contributing to the degradation of proteins and organelles. Protein synthesis is impaired, exacerbating muscle loss. Apoptotic signals are also involved: Increased pro-apoptotic protein Bax and decreased anti-apoptotic protein BcL-2 promote cell death in muscle tissue. Fast-twitch muscle fibers are particularly vulnerable to oxidative stress, which is accentuated by mitochondrial dysfunction. Damaged mitochondria produce less ATP and more reactive oxygen species (ROS), setting in motion a pathological cycle that amplifies cellular damage, protein degradation, and muscle atrophy. In AD, pathological accumulation of β-amyloid peptide (Aβ), oxidative stress and mitochondrial dysfunction are central elements in neurodegeneration. Impaired amyloid precursor protein (APP) cleavage leads to the formation of toxic Aβ aggregates, which contribute to synaptic dysfunction and neuronal death. Mitochondrial dysfunction reduces ATP production and increases ROS production, fueling a state of chronic oxidative stress that damages cells and activates apoptotic pathways. In addition, Aβ accumulation is observed at the muscle level, which induces endoplasmic reticulum (ER) dysfunction, mitochondrial alterations and activation of apoptosis. Finally, in PD, in addition to the degeneration of dopaminergic neurons, systemic processes involving skeletal muscle are observed. Oxidative stress and mitochondrial dysfunction are central to the pathogenesis: Damaged mitochondria produce less ATP and increase ROS, damaging lipids, proteins and DNA. Mitochondrial abnormalities, reduced complex I activity and altered oxidative phosphorylation are found in the muscles of PD patients. Furthermore, reduced expression of key proteins for the release of Ca^2+^ from the sarcoplasmic reticulum (such as ryanodine receptors, calsequestrin-1 and triadin) impairs muscle contractility, contributing to bradykinesia and tremor. In peripheral nervous tissue, an increase in inflammatory cytokines (TNF-α, IL-1β and IL-6) and an infiltration of macrophages and T lymphocytes into the muscle are recorded, a sign of chronic inflammation that promotes muscle degeneration and aggravates motor symptoms, indicating that the muscle is an active player in the progression of the disease. 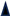 = increase 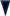 = decrease.

The role of muscle tissue in the pathophysiology of PD has become an area of increasing research focus in recent years. Emerging studies suggest that muscle tissue not only experiences secondary changes due to neural degeneration but may also play an active role in disease progression and the manifestation of symptoms. In individuals with PD, muscle tissue exhibits a variety of structural and functional alterations [98]. These changes are primarily linked to decreased neural input resulting from the degeneration of dopaminergic neurons, which in turn leads to motor dysfunction. Histopathological examinations of muscle biopsies from PD patients have revealed distinct changes, such as muscle fiber atrophy, an increased prevalence of type II fibers, and noticeable mitochondrial abnormalities. Atrophy of muscle fibers, particularly in the type II (fast-twitch) fibers, is a common finding, likely a consequence of denervation and disuse due to motor impairments in PD. Additionally, there is a shift toward a higher proportion of type I (slow-twitch) fibers [107,108]. This change in muscle fiber composition may represent an adaptive mechanism in response to the chronic nature of PD, which prioritizes endurance over rapid, high-intensity contractions. Muscle biopsies from PD patients often show an increased presence of dysfunctional mitochondria, including mitochondrial swelling, disruption of cristae structures, and the accumulation of electron-dense material [109]. Recent research has indicated that the quadriceps femoris muscles in the MPTP-induced mouse model of PD exhibit a diminished expression of key proteins involved in sarcoplasmic reticulum (SR) Ca^2+^ release, including ryanodine receptors (RyR), calsequestrin 1, and triadin [110]. This reduced protein expression disrupts the release of Ca2^+^ from the sarcoplasmic reticulum, leading to impaired muscle contraction dynamics, including decreased force and velocity, which contribute to symptoms such as bradykinesia and tremor in PD patients. Chronic, excessive cytosolic Ca^2+^ release, commonly observed in PD, is believed to play a significant role in the dysfunction of cellular structures such as the endoplasmic reticulum, mitochondria, and lysosomes within the brain. Approaches targeting the regulation of Ca^2+^ handling proteins to prevent cellular Ca^2+^ overload could potentially mitigate neurodegenerative processes and oxidative damage and improve the force and speed of muscle contractions in individuals with PD. Mitochondrial dysfunction has been widely recognized as a crucial factor in the development of PD, influencing both central nervous system and peripheral tissues such as skeletal muscle [109]. In the context of PD muscle tissue, mitochondrial abnormalities are characterized by a decline in the activity of the respiratory chain, elevated oxidative stress, and the presence of genetic mutations [111]. Muscle biopsies obtained from PD patients reveal a decrease in the activity of mitochondrial respiratory chain complex I that compromises oxidative phosphorylation, impairing ATP production, which is essential for cellular function [21]. In addition, mitochondrial dysfunction in PD is associated with an increase in the generation of ROS, which contributes to oxidative damage in various cellular components, such as lipids, proteins, and DNA. This oxidative stress not only exacerbates mitochondrial dysfunction but also accelerates muscle tissue degeneration and dysfunction [112,113]. The ongoing damage to cellular structures due to ROS accumulation further amplifies the pathological processes, creating a vicious cycle that contributes to both neurodegeneration and muscular impairments seen in PD. Recent research has revealed the presence of α-synuclein aggregates in the muscle tissue of individuals with PD. These aggregates are believed to disrupt cellular balance, promoting muscle dysfunction through mechanisms such as impaired protein degradation, cytotoxic effects, and disrupted neuronal–muscular interactions [114]. Finally, studies have shown elevated levels of pro-inflammatory cytokines, such as TNF-α, IL-1β and IL-6, in the sural nerves of PD patients, which can worsen muscle degeneration and amplify disease severity [115,116]. Additionally, immune cells like macrophages and T lymphocytes are more frequently found in PD muscle tissue, potentially triggered by damage-associated molecular patterns (DAMPs) released from damaged muscle cells [117]. The chronic systemic inflammation observed in PD can negatively affect muscle tissue, promoting catabolic processes and contributing to muscle wasting (Figure 3).

## 5. Conclusions

Although neurodegenerative disorders exhibit a wide range of symptoms, a common feature among these diseases is the presence of oxidative stress. This imbalance affects not only neuronal cells but also muscle cells. Skeletal muscle, as a peripheral tissue, is among the most affected in neurodegenerative diseases, actively contributing to pathogenesis and clinical deterioration.

The temporal onset of muscular symptoms, including oxidative stress, remains a subject of debate. In this context, various lines of evidence suggest that redox alterations in skeletal muscle may precede disease onset, positioning oxidative stress as both a predictive and prognostic biomarker.

Overall, this review outlines the role of oxidative stress in skeletal muscle in the context of neurodegenerative diseases, focusing on its implications for pathogenesis, prognosis, and therapeutic strategies. Indeed, there is growing interest in treatments targeting ROS imbalance while also promoting proper mitochondrial function.

## Figures and Tables

**Table 1:** Summary of key enzymes involved in ROS balance.

Enzyme	Classification	EC Number	Main Substrate(s)	Product(s)	Cellular Role
NADPH oxydase (NOX)	pro-oxidant	EC 1.6.3.1	NADPH + O_2_	Superoxide (O_2_•^−^) + NADP^+^ + H^+^	Host defense, signaling, and Cell differentiation
Myeloperoxidase (MPO)	pro-oxidant	EC 1.11.2.2	H_2_O_2_ + Cl^−^ (or Br^−^)	Hypochlorous (or hypobromous acid) acid (HOCl, HOBr) + H_2_O	Antimicrobial activity
Xanthine oxidase (XOR)	pro-oxidant	EC 1.17.3.2	Hypoxanthine/Xanthine + O_2_	Uric acid + Superoxide (O_2_•^−^·) + Hydrogen peroxide H_2_O_2_	Catabolism of purine nucleotides
Superoxide dismutases (SODs)	anti-oxidant	EC 1.15.1.1	Superoxide (O_2_•^−^)	Hydrogen peroxide H_2_O_2_ + O_2_	ROS detoxification, Antioxidant defense, Redox signaling modulation, Cell survival and homeostasis, Inflammation regulation
Catalase (CAT)	anti-oxidant	EC 1.11.1.6	Hydrogen peroxide (H_2_O_2_)	Water (H_2_O) + O_2_	Antioxidant defense, Redox homeostasis, Redox signaling modulation, Cell survival, Inflammation control

## Data Availability

Data are available from the authors upon reasonable request.

## Abbreviations

The following abbreviations are used in this manuscript: Alzheimer’s disease (AD); Amyloid precursor protein (APP); Amyloid-β (Aβ); Amyotrophic lateral sclerosis (ALS); Autophagy–lysosome pathway (ALP); BCL2/adenovirus E1B 19 kDa protein-interacting protein 3 (BNIP3); Branched-chain amino acids (BCAAs); Catalase (CAT); Copper and Zinc (Cu/Zn); Damage-associated molecular patterns (DAMPs); Dehydrogenase (DH); Dual Oxidases (DUOX1, DUOX2); Dynamin-1-like protein (Drp1); Endoplasmic reticulum (ER); Extracellular signal-regulated kinases (ERK); Histidine (His); Hydrogen peroxide (H_2_O_2_); Hydroxyl radicals (•OH); Hypobromous acid (HOBr); Hypochlorous acid (HOCl); Hypoxia-inducible factor-1α (HIF1α); Interleukin-1β (IL-1β); Interleukin-6 (IL-6); Intracellular calcium (Ca^2+^); Lipopolysaccharides (LPS); Magnetic resonance (MR); Manganese (Mn); Mitofusin 1, 2 (MFN1, MFN2); Mitogen-activated protein kinase (MAPK); Molybdenum (Mo); Myeloperoxidase (MPO); Myosin heavy chain (MyHC); N-acetylaspartate (NAA); N-acetylcysteine (NAC); Neuromuscular junctions (NMJs); Nicotinamide adenine dinucleotide phosphate oxidase or NADPH oxidase (NOX); Nuclear factor of activated T-cells (NFAT); Parkinson’s disease (PD); Peroxisome proliferator-activated receptor gamma coactivator 1-alpha (PGC-1α); Reactive nitrogen species (RNS); Reactive oxygen species (ROS); Ryanodine receptors (RyR); Short-chain fatty acids (SCFAs); Superoxide anion radicals (O_2_•^−^); Superoxide dismutases (SODs); Toll-like receptor 4 (TLR4); Transmembrane (TM); Tumor necrosis factor (TNF-α); Two non-identical iron-sulfur centers (2Fe/S); Ubiquitin–proteasome system (UPS); Xanthine oxidase (XOR); α-synuclein (α-syn).
